# Research on fault diagnosis method for variable condition planetary gearbox based on SKN attention mechanism and deep transfer learning

**DOI:** 10.1038/s41598-025-04858-9

**Published:** 2025-07-02

**Authors:** Nai-Qiu Huang, Meng-Meng Song, Yao-Hong Tang, Li-Xia Huang, Zhi-Wen Chen

**Affiliations:** 1 College of Mechanical and Electrical Engineering, Ningde Normal University, Ningde, China; 2 Department of Nature Science and Computer, Ganzhou Teachers College, Gan Zhou, China

**Keywords:** Electrical and electronic engineering, Mechanical engineering

## Abstract

Deep learning network models are widely applied to fault diagnosis of planetary gearboxes. However, the multi-coupling fault characteristics, accompanied by data fuzziness and distribution differences, present certain challenges to diagnostic research. Under variable operating conditions, the fault data to be diagnosed becomes more prominently inconsistent in distribution, leading to suboptimal fault recognition rates in diagnostic models. A deep transfer learning method for planetary gearbox fault diagnosis based on a Selective Kernel Networks (SKN) attention mechanism is proposed. First, an input dataset is constructed through overlapping sampling, and a deep neural network diagnostic model is established to automatically learn features and perform diagnostics on the data. Second, a dynamic selection mechanism for convolution kernels is embedded in the deep neural network, enabling each neuron to adaptively adjust its receptive field size based on multi-scale input information. This mechanism extracts common features between the source and target domains, enhancing the network’s feature extraction capability. Third, the Local Maximum Mean Discrepancy (LMMD) is used to perform sub-domain adaptation on the features of the source and target domains, reducing the distribution discrepancy between the two domains and constructing an end-to-end transfer adaptation model. This enables deep transfer learning fault diagnosis of planetary gearboxes under varying operating conditions. Finally, through experimental analysis and validation of 8 variable operating condition tasks, the fault identification accuracy of the diagnostic method proposed in this paper reached an average of 92.9%. Compared with traditional deep transfer learning diagnostic methods, it demonstrates higher diagnostic precision.

## Introduction

Clean energy, characterized by its low carbon and environmentally friendly attributes, is being widely advocated and researched. Wind power generation, as a representative of clean energy, is rapidly being developed and applied worldwide^1^. Planetary gearboxes, known for their compact structure, high load capacity, smooth transmission^2^, and ability to achieve large transmission ratios in small spaces^3^, are widely used as essential components in wind turbines. However, due to the varying characteristics of workload, rotational speed, and environmental conditions, critical components of planetary gearboxes, such as the sun gear, planet gears, and planet carrier, often experience issues like tooth surface pitting, wear, adhesion, and tooth breakage^4^. If these faults are not detected in time, they can further escalate, potentially leading to equipment damage or even major accidents^5^. Therefore, real-time fault diagnosis of planetary gearboxes in wind turbines is of great significance to ensure their normal operation^6^.

The fault diagnosis method for planetary gearboxes involves three crucial steps: acquiring vibration signals, extracting effective fault features, and evaluating and classifying faults^7^. In traditional fault diagnosis, signal acquisition and analysis are often used to assess the health status of planetary gearboxes. Time-domain analysis involves statistical processing of signals^8^, while frequency-domain analysis employs Fourier Transform (FFT) to convert vibration signals from the time domain to corresponding frequency components and amplitudes^9^. Time–frequency domain analysis combines both time and frequency domain analyses to obtain fault feature information of the gearbox^10^. Although signal analysis and processing techniques have proven effective for diagnosing faults in planetary gearboxes, relying solely on acquired signals may not fully reflect fault information, which can affect diagnostic accuracy. Machine learning fault diagnosis methods extract features from signal analysis, label samples, and input labeled features into machine learning models to establish a correspondence between operating conditions and fault characteristics, completing the diagnosis. Machine learning methods such as K-Nearest Neighbors (KNN), Support Vector Machine (SVM), and Artificial Neural Networks (ANN) still require feature extraction and demand substantial domain knowledge and expertise. Additionally, manual feature extraction depends on prior evaluation criteria, making it challenging to effectively uncover new fault features.

In recent years, with the development of deep learning algorithms in artificial intelligence, the advantages of feature learning through deep neural networks have been widely applied in fault diagnosis. Chen et al.^11^ utilized convolutional neural networks to identify fault types in planetary gearboxes by extracting time-domain and frequency-domain features of the gearbox. Wang et al.^12^ proposed a multi-tasking atrous convolutional neural network (MACNN) in order to achieve various and complex rotating machinery fault diagnoses. Jiang et al.^13^ used a fault diagnosis method based on local bi-spectrum and CNN to realize the classification diagnosis of different fault positions and degrees of planetary gearboxes. Chen et al.^14^ developed an adaptive neural network model based on real-time rotational speed conditions to achieve fault classification of planetary gearboxes under various operating environments. Liu et al.^15^ designed a novel algorithm for bearing fault diagnosis using sparse wavelet decomposition for feature extraction combined with a multi-scale one-dimensional convolutional neural network (1-D CNN), which achieved a higher classification accuracy on rolling bearings. Miao et al.^16^ proposed an improved intelligent fault detection method for rotating component based on interactive channel attention (ICA), and a multi-scale convolutional layer with an adaptive selective kernel (ASK) unit is applied to replace the basic convolutional layer of CNN, which is superior to the comparison methods,especially in the occasions with strong noise and limited data.

The above methods are based on the assumption that the training data and test data follow independent and identically distributed conditions in order to achieve fault recognition. However, in real industrial scenarios, ensuring that the training samples and test samples have the same feature distribution is extremely challenging. This not only requires considerable effort but is also difficult to accomplish.

Transfer learning enables the knowledge acquired from different but related domains to be transferred to a target domain, facilitating the recognition of target tasks^17^. Chen et al.^18^ employed a pre-trained convolutional neural network using source domain samples and fine-tuned the network with a small number of labeled target domain samples to achieve fault diagnosis in the target domain. Yang et al.^19^ developed a feature transfer fault diagnosis model that learned fault characteristics from experimental data to assess the health status of locomotives. Han et al.^20^ proposed a method that simultaneously conducts supervised classification and multiple adversarial domain adaptation models to address the issue of data sparsity. Du et al.^21^ revealed a method based on an improved MobileNetV3 network and transfer learning (TL-Pro-MobileNetV3 network). The experimental results showed the accuracy of the method proposed can reach 100% and the training time was the shortest in two working conditions in gearbox fault diagnosis. Han et al.^22^ presented a framework can exploit the discrimination structures associated with the labeled data in source domain to adapt the conditional distribution of unlabeled target data, and thus guarantee a more accurate distribution matching, by extending the marginal distribution adaptation (MDA) to joint distribution adaptation (JDA). Extensive empirical evaluations on three fault datasets validate the applicability and practicability of DTN. Yao et al.^23^ proposed a deep transfer convolutional neural network (DTCNN), which was trained with both source and target data, and a fine-tuning strategy was employed to effectively eliminate distribution discrepancies between different battery types or charging/discharging protocols. Experimental resulted demonstrate that the proposed method accurately estimates the lithium-ion battery capacity.

The aforementioned method involves training basic deep convolutional neural networks and designing various global domain adaptation strategies to reduce the discrepancies between training data and real-time monitoring test data. It also focuses on learning shared features between them to achieve cross-domain diagnostic evaluation for different types of machinery. However, given the complex characteristics of multi-coupled fault samples in planetary gearboxes, merely focusing on global domain adaptation methods under various fault conditions no longer meets diagnostic requirements.

To address the issue of sample distribution changes caused by load and speed variations in the practical application of planetary gearboxes, this paper proposes a fault diagnosis method suitable for varying working conditions. This method effectively amplifies the multi-coupled fault characteristic information of planetary gearboxes while suppressing the background information of working conditions. It aligns the distribution of similar fault subdomains across different domains to achieve accurate fault classification under limited sample conditions. The contributions of the fault diagnosis method proposed in this paper are as follows:

1. The SKN attention mechanism is embedded before the fully connected layer of the convolutional neural network. Experiments comparing it with other mainstream attention mechanisms indicate that the SKN attention mechanism can adaptively adjust the receptive field to enhance features with fault discrimination while suppressing features sensitive to changes in working conditions.
2. In transfer learning, LMMD is used to measure the distribution differences between fault subdomains of the source and target domains, considering the relationships between subdomains within the same category but different domains. It measures the differences between fault state subdomains in different working condition domains and aligns the distribution of related subdomains. This approach not only aligns the global distribution of source and target domains but also integrates deep feature learning and feature adaptation within an end-to-end deep learning model to align the distribution of related subdomains.

The rest contents of this paper are as follows. In section “Theoretical background”, the paper primarily introduces the theory of the SKN attention mechanism and the LMMD principle. In section “The proposed fault diagnosis model process”, mainly discusses the framework structure and workflow of the proposed fault diagnosis model. In section “Experimental analysis and validation”, presents the experimental setup, compares the impact of different attention mechanisms on the transfer model, and provides a detailed analysis of the differences between various deep learning models and the proposed improved DSAN model (SK-DSAN model) in diagnosing faults in planetary gearboxes. Conclusions are presented in Section “Conclusions”.

## Theoretical background

### Domain adaptation theory

The purpose of transfer learning is to transfer the knowledge learned from a labeled source domain dataset to an unlabeled target domain dataset. During the transfer process, the model adaptively adjusts the parameters learned from the source domain and generalizes them to apply to unknown target domain tasks.

*D*_*s*_ and *D*_*t*_ are collected from different data distributions under the probabilities p and q, respectively, where p ≠ q, and the two domains may have different categories $y_{i}^{s} \ne y_{i}^{t}$. Due to the differences in distribution and categories between the two domains, a model learned and trained from the source domain cannot be directly used for task diagnosis in the target domain. In deep neural networks, the feature mapping functions for the source domain and the target domain are, respectively:

1 $$O^{s} = L^{s} (x^{s} ,y^{s} ;\theta^{s} )$$

2 $$O^{t} = L^{t} (x^{t} ,y^{t} ;\theta^{t} )$$

*O*^*s*^ and *O*^*t*^ represent the network outputs for the source domain and target domain, respectively, while $L^{s} \left( . \right)$ and $L^{t} \left( . \right)$ denote the network feature mapping functions for the source and target domains. Transfer learning establishes correspondences by mapping the network weights and bias parameters *θ*^*s*^ learned in the source domain to the network weights and bias parameters *θ*^*t*^ in the target domain. During this process, domain adaptation adjusts the parameters and generalizes classification within the target domain.

### Convolutional Neural Networks

A Convolutional Neural Network (CNN) is a typical multi-layer deep learning neural network model. It can achieve abstract representation by extracting low-level local features, sharing network layer parameters, and reducing the number of parameters needed for training^24^. A CNN typically consists of convolutional layers, activation functions, pooling layers, and fully connected layers.

### SKN attention mechanism

**Fig. 1:**
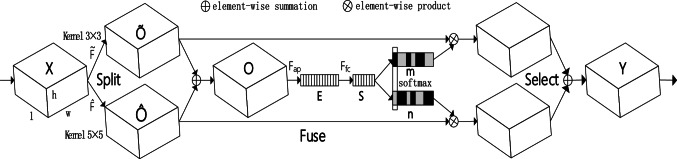
Selective Kerne.

Selective Kernel Networks (SKN) channel attention mechanism is essentially similar to the human selective visual attention mechanism. Its core purpose is to filter out crucial and useful information for the current task from the learned information. To enable neurons to adaptively adjust their receptive field (RF) size among kernels of different sizes, an automatic selection operation called “Selective Kernel” (SK) convolution is proposed^25^. This is achieved through three operational steps: Split, Fuse, and Select, as illustrated in Fig. 1. The Split operator can generate multiple paths with different kernel sizes. In this model, only two convolution kernels of different sizes are designed, but it can actually be extended to multiple branches with multiple convolution kernels. The Fuse operator combines and aggregates information from multiple paths to obtain a global and comprehensive representation for selecting weights. The Select operator aggregates features of different kernel sizes based on the selection weights.

Split: The input feature map is denoted as $X \in R^{H^{\prime} \times W^{\prime} \times L^{\prime}}$, which undergoes two transformations: $\tilde{F}:X \to \tilde{O} \in R^{H \times W \times L}$ and $\hat{F}:X \to \hat{O} \in R^{H \times W \times L}$, with convolution kernel sizes of 3 × 3 and 5 × 5, respectively. $\tilde{F}$ and $\hat{F}$ are processed using efficient group or depthwise convolutions, followed by batch normalization and the ReLU activation function in sequence. To improve training efficiency, the 5 × 5 convolution kernel is replaced with a 3 × 3 convolution kernel with a dilation rate of 2.

Fuse: To enable neurons to adaptively adjust the size of their receptive fields based on stimuli, multiple branches are used through channels to carry information of different scales to the next layer of neurons. Taking Fig. 1 as an example, it illustrates the result of fusing two branches.

3 $$O = \tilde{O} + \hat{O}$$

To embed global information, global average pooling is used to generate the channel statistics of $E \in R^{c}$.This is done by computing the *c*-th element of *E* through the spatial dimensions *H* × *W*, reducing *O* in the process.

4 $$E_{c} = F_{ap} \left( {O_{c} } \right) = \frac{1}{H \times W}\sum\limits_{i = 1}^{H} {\sum\limits_{j = 1}^{W} {O_{c} \left( {i,j} \right)} }$$

The connection layer creates a compact feature $S \in R^{d \times 1}$.

5 $$S = F_{fc} \left( E \right) = \delta \left( {{\rm B}\left( {G_{E} } \right)} \right)$$

In this context, *δ* represents the ReLU activation function, and *B* denotes batch normalization, $G \in R^{d \times c}$.

Select: Apply the softmax function to the channel numbers, and then perform multiplication and summation operations with the features obtained after the split convolution.

6 $$m_{c} = \frac{{e^{{M_{c} S}} }}{{e^{{M_{c} S}} + e^{{N_{c} S}} }},\quad n_{c} = \frac{{e^{{N_{c} S}} }}{{e^{{M_{c} S}} + e^{{N_{c} S}} }}$$

In this context $M,N \in R^{c \times d}$, *m* and *n* represent the *d*-dimensional soft attention vectors of $\tilde{O}$ and $\hat{O}$, respectively, while $M_{c} \in R^{1 \times d}$ denotes the *c*-th row of vector *M*, which is also its *c*-th element. When there are only two branches, *m*_*c*_ + *n*_*c*_ = 1, making matrix *N* redundant. The final feature *Y* is obtained by aggregating the kernel features through the convolutional kernel attention weights on each branch.

7 $$Y_{c} = m_{c} .\tilde{O}_{c} + n_{c} .\hat{O}_{c} ,\quad m_{c} + n_{c} = 1$$

of which $$Y = \left[ {Y_{1} ,Y_{2,} ...,Y_{c} } \right]$$,$$Y_{c} \in R^{H \times W}$$.

### Local Maximum Mean Discrepancy

**Fig. 2:**
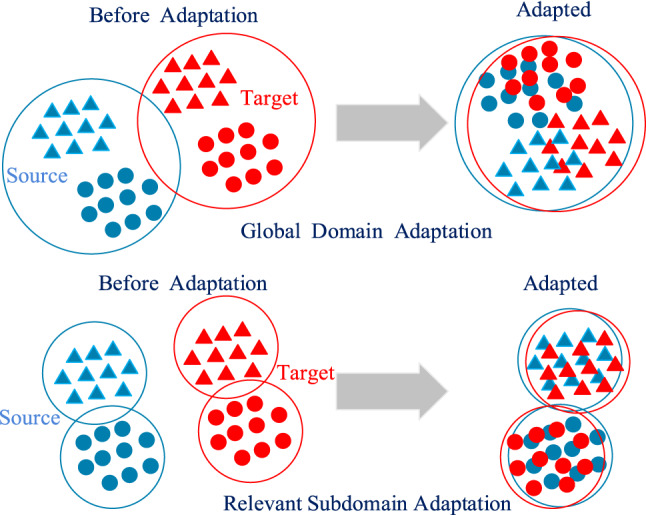
Adaptation principles of domain and subdomain.

Maximum Mean Discrepancy (MMD) is a non-parametric distance measure used to evaluate the difference between the distributions of the source and target domains. Domain adaptation primarily focuses on aligning the global domain distribution, often overlooking the relationship between intra-class subdomains within the global domain. This oversight can hinder the capture of more granular information during transfer learning, resulting in suboptimal domain adaptation performance^26^. To address this issue, this paper introduces Local Maximum Mean Discrepancy (LMMD), which aligns the mappings of each subdomain in the source and target domains. This approach matches the local distribution of feature samples from the source domain to the target domain, while also achieving global distribution alignment, thereby obtaining invariant features across the global domain. The principles of domain and subdomain adaptation are illustrated in Fig. 2.

In the given source domain $D_{S} = \left\{ {\left( {x_{i}^{s} ,y_{i}^{s} } \right)} \right\}_{i = 1}^{{n_{s} }}$ with *n*_*s*_ labeled samples and target domain $D_{t} = \left\{ {x_{j}^{t} } \right\}_{j = 1}^{{n_{t} }}$ with *n*_*t*_ unlabeled samples, $y_{i}^{s}$ represents the one-hot label corresponding to the *i*th source domain sample $x_{i}^{s}$, $y_{i}^{s} = c$ indicates that a sample belongs to the *c*th class, $x_{j}^{t}$ is the *j*th unlabeled target domain sample, and the LMMD calculation formula is as follows:

8 $$\hat{d}_{H} \left( {p,q} \right) = \frac{1}{C}\sum\limits_{c = 1}^{C} {\left\| {\sum\limits_{{x_{i}^{s} \in D_{s} }} {w_{i}^{sc} \varphi (x_{i}^{s} ) - \sum\limits_{{x_{j}^{t} \in D_{t} }} {w_{j}^{tc} \varphi (x_{j}^{t} )} } } \right\|}_{H}^{2}$$

*P* and *q* represent the sample distributions of the source domain and target domain, respectively. *H* is a Reproducing Kernel Hilbert Space (RKHS) with a feature kernel. *φ(.)* denotes a feature mapping that can map the original sample data to *H*. $x_{i}^{s}$ and $x_{j}^{t}$ are the *i*th and *j*th samples from the source and target domains, respectively. $w_{i}^{sc}$ and $w_{j}^{tc}$ are the weights of these samples belonging to category *c*. The values of $\sum {_{i = 1}^{{n_{s} }} } w_{i}^{sc}$ and $\sum {_{j = 1}^{{n_{t} }} } w_{j}^{tc}$ are 1. The weight $x_{i}^{{}}$ corresponding to sample $w_{i}^{c}$ can be determined by the following equation:

9 $$w_{i}^{c} = \frac{{y_{ic} }}{{\sum {_{{(x_{j} ,y_{j} ) \in D}} y_{jc} } }}$$

$y_{ic}^{{}}$ is the *c*-th element of vector $y_{i}^{{}}$. For samples in the source domain, we use the true label $y_{i}^{s}$ as a one-shot vector to calculate the weight $w_{i}^{sc}$ for each sample. In unsupervised adaptation for the target domain with unlabeled samples, $y_{j}^{t}$ is unavailable, making it impossible to compute distribution differences. The output of the deep neural network $\hat{y}_{i} = f(x_{i} )$ is a probability distribution representing the probability of recognizing $x_{i}^{{}}$ as class *c*. Thus, the results predicted by the network model can be used as pseudo-labels to calculate the weight $w_{j}^{tc}$ of the target domain, measure distribution differences, and complete model training.

Given a source domain *D*_*s*_ with *n*_*s*_ labeled samples and a target domain *D*_*t*_ with *n*_*t*_ unlabeled samples, each following independent and identically distributed distributions *p* and *q* respectively, the deep neural network generates activations *I* and *J* at the *l*-th layer. Since *φ(.)* cannot be directly computed, the distribution discrepancy function is re-expressed as:

10 $$\hat{d}_{l} (p,q) = \frac{1}{C}\sum\limits_{c = 1}^{C} {\left[ {\sum\limits_{i = 1}^{{n_{s} }} {\sum\limits_{j = 1}^{{n_{s} }} {w_{i}^{sc} w_{j}^{sc} k(z_{i}^{sl} ,z_{j}^{sl} ) + \sum\limits_{i = 1}^{{n_{t} }} {\sum\limits_{j = 1}^{{n_{t} }} {w_{i}^{tc} w_{j}^{tc} k(z_{i}^{tl} ,z_{j}^{tl} ) - 2\sum\limits_{i = 1}^{{{n_{s}{}} }} {\sum\limits_{j = 1}^{{n_{t} }} {w_{i}^{sc} w_{j}^{tc} k(z_{i}^{sl} ,z_{j}^{tl} )} } } } } } } \right]}$$

In the formula, *z*^*l*^ represents the activation of layer *l* in (*l* ∈ *L* = {1,2,…,|*L*|}). Most feedforward neural network models can achieve LMMD, thereby enabling subdomain adaptation.

## The proposed fault diagnosis model process

**Fig. 3:**
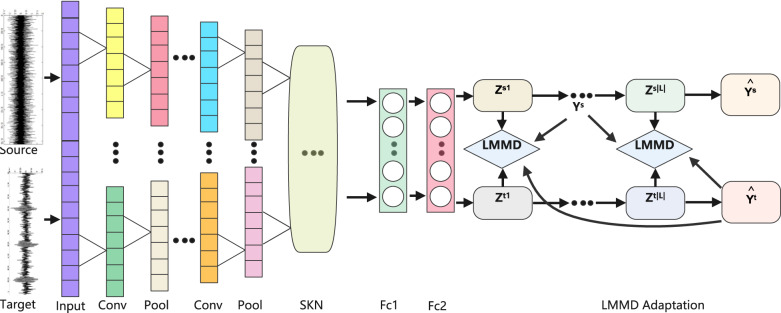
Structure of fault diagnosis model for the proposed method.

Under variable operating conditions, the planetary gearbox fault diagnosis model based on the SKN convolutional neural network deep subdomain adaptation method is shown in Fig. 3.

The raw vibration signal is input into the convolutional layer, where multiple convolution filters perform convolution operations to extract local features of the original vibration signal. A batch normalization layer is added to normalize the features of the training data, reducing the variance in feature learning across layers. The max pooling layer selects the maximum value from local regions of the input data as the feature output, ensuring translation invariance is obtained from the input features. Following the max pooling layer, a Dropout layer is added to reduce interdependence in feature learning among nodes.

The SKN channel attention mechanism suppresses useless features and amplifies effective features, integrating weight information to further extract signal characteristics. The SKN channel module is a key component for improving the model’s adaptability to operating conditions. It enhances channel features that are more sensitive to the health state of the planetary gear and suppresses channel features that are sensitive to operating condition changes. The fault characteristics of the planetary gearbox are manifested as vibration acceleration during the engagement process between the planet gears and the ring gear, as well as the sun gear. This vibration acceleration is particularly noticeable at the moments when the planet gears engage with the ring and sun gears, which will serve as the distinguishing characteristic to differentiate fault types. The fault vibration signal features extracted through convolutional pooling operations are first processed by SKN with multi-branch separable convolutions. Each branch uses different convolution kernels (receptive fields) for feature extraction, with features distinguishing fault types receiving greater attention. The feature extraction results from the multiple branches are then summed, and global average pooling is applied for dimensional transformation, as shown in Eq. (4). The feature dimension is first reduced using a fully connected layer, as shown in Eq. (5), and then the dimensionality is restored to the same level as before the reduction to obtain the feature vector. The feature vector is then processed through a softmax function, as shown in Eq. (6). The feature vector processed by softmax is multiplied by the multi-branch extracted features before transformation. Finally, the n feature vectors are summed together. As shown in Eq. (7), when n = 2, the greater the weight of the fault feature vector, the more prominent the output feature vector. Under varying operating conditions, the fault feature vector is similarly enhanced, though the background baseline vibration value changes. In this way, SKN enhances features that are discriminative for fault diagnosis and suppresses irrelevant features.

Unlike the majority of deep domain adaptation methods, which focus on aligning the global distributions of the source and target domains without considering the relationships between subdomains within the same category across different domains, these methods derive the global domain shift between the source and target domains. After adaptation, the global distributions of both domains are approximately identical. However, global alignment may result in irrelevant data becoming too similar to one another, making accurate classification difficult. This model aligns the distributions of relevant subdomains by measuring the differences between fault state subdomains under different operating conditions. It not only matches global distributions but also aligns local distributions. The model not only aligns the global distributions of the source and target domains but also integrates deep feature learning and feature adaptation within an end-to-end deep learning model to align the distributions of relevant subdomains. Subdomain adaptation can capture fine-grained information for each fault category. Considering different sample weights, the Hilbert–Schmidt norm between the kernel mean embeddings of the empirical distributions of related fault subdomains in the source and target domains is measured, thereby achieving proper alignment. The difference in activations between the distributions of related fault subdomains in layer L is reduced, with the subdomain adaptation loss for a specific L layer expressed using the LMMD expression in Eq. (10):

11 $$\mathop {\min }\limits_{f} \frac{1}{{n_{s} }}\sum\limits_{i = 1}^{{n_{s} }} {J\left( {f(x_{i}^{s} ),y_{i}^{s} } \right)} + \lambda \sum\limits_{l \in L} {\mathop d\limits^{ \wedge }_{l} } (p,q)$$

J(., .) is the cross-entropy loss function, also known as the classification loss function, while ${\hat{\text{d}}}$(., .) represents the domain adaptation loss function. The parameter λ > 0 serves as a balancing factor between the domain adaptation loss function and the classification loss function. The model is trained and fine-tuned following the standard mini-batch stochastic gradient descent algorithm. After iterations, the fault classification of the target domain samples becomes more accurate. The LMMD adaptive metric criterion is used to match the conditional distribution differences between the source domain and the target domain in the network adaptation layer, and it is fully connected to output the probability of the category to which each input sample belongs. By utilizing the subdomain adaptation function and the cross-entropy loss function, the gradient backpropagation algorithm is employed to update the network parameters. The main parameters of the proposed model’s network structure are shown in Table 1.

**Table 1:** Proposed model network structure parameters.

Network structure	Convolution kernel size	Number of convolution kernels	Output size
Convolution layer1	64 × 1	16	128 × 16
Pooling layer1	2 × 1	16	64 × 16
Convolution layer2	3 × 1	32	64 × 32
Pooling layer2	2 × 1	32	32 × 32
Convolution layer3	3 × 1	64	32 × 64
Pooling layer3	2 × 1	64	16 × 64
Convolution layer4	3 × 1	64	16 × 64
Pooling layer4	2 × 1	64	8 × 64
Convolution layer5	3 × 1	64	6 × 64
Pooling layer5	2 × 1	64	3 × 64
SKN layer	–	–	3 × 64
Full connection layer 1	hidden_size	–	hidden_size
Full connection layer 2	–	3	3 × 1

The main steps of the proposed model for fault diagnosis are as follows:

Collect samples with known fault information under a specific operating condition as the source domain sample set. The source domain sample set is divided into a training sample set and a test sample set. Samples with unknown fault information from other operating conditions form the target domain sample set, which includes both the target domain training sample set and the test sample set, thereby constructing datasets for different operating conditions.

Utilize the proposed model to obtain distributed feature representations of both the source and target domains. Introduce the SKN channel attention mechanism to focus on channel selection, filtering out key useful information from the learned data.

Calculate the local maximum mean discrepancy between the source and target domains using the deep features obtained from both domains, along with the true labels from the source domain and pseudo labels from the target domain. Employ the LMMD adaptive measurement criterion to match the distribution differences between subdomains of the source and target domains, reducing subdomain distribution differences under varying operating conditions. Through iterative training, the model is obtained.

Input the target domain test sample set and use the softmax classification layer to output diagnostic results, completing the fault diagnosis of the planetary gearbox under varying operating conditions.

## Experimental analysis and validation

### Experimental parameter data

**Fig. 4:**
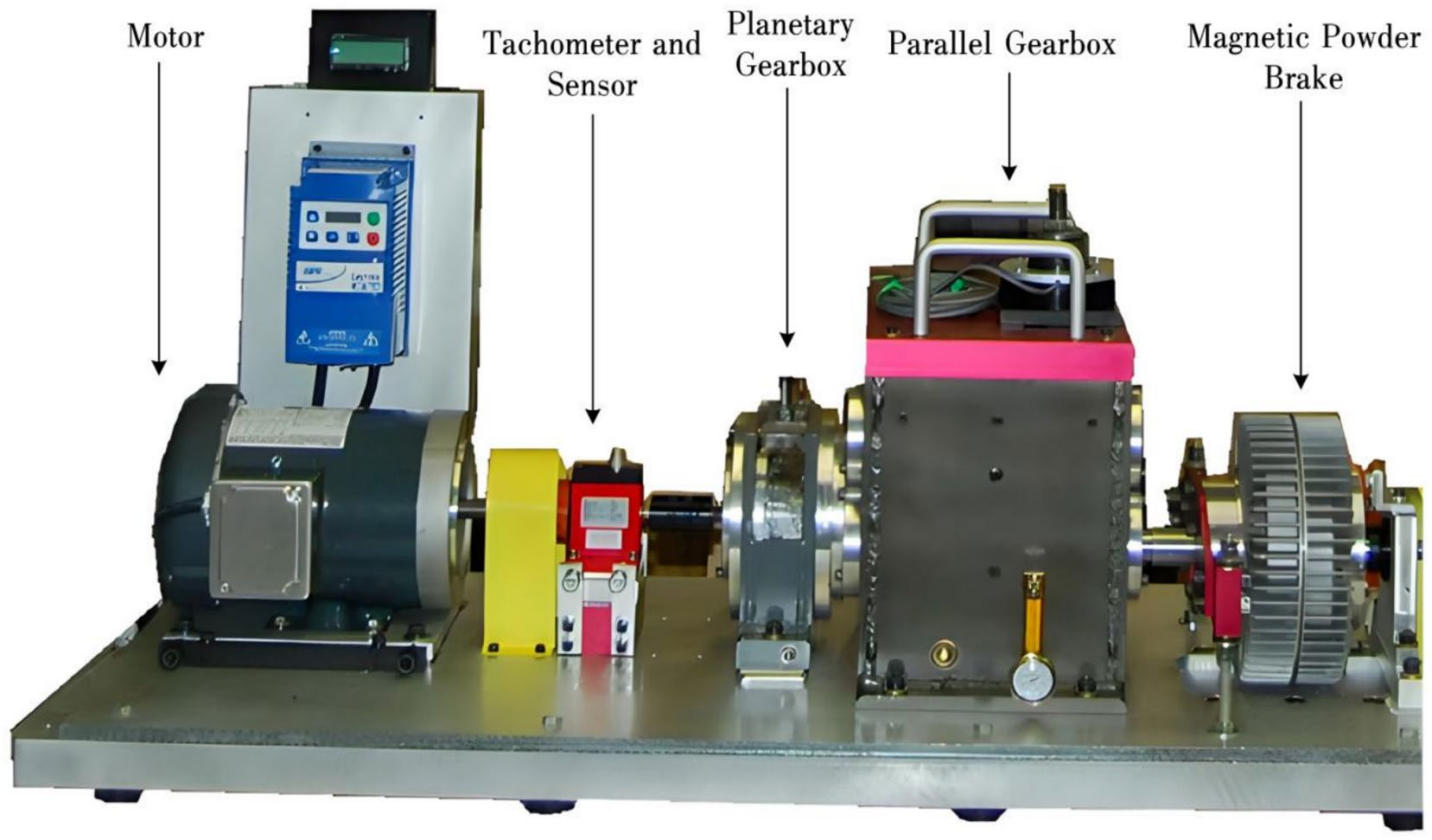
DDS planetary gearbox fault diagnosis experimental platform.

To verify the superiority and effectiveness of the proposed SKN-based deep transfer model, experiments were conducted using the DDS planetary gearbox fault diagnosis experimental platform. The setup is shown in Fig. 4. The speed of the variable-speed drive motor is controlled by a frequency converter, with the frequency set at 20 Hz, 30 Hz, and 40 Hz to control the rotational speed. A magnetic brake simulates the load on the transmission mechanism, with currents of 0 A, 0.4 A, and 0.8 A applied to the magnetic powder brake to simulate three load conditions. For each motor speed, the magnetic powder brake connected to the planetary gearbox transmission mechanism can be supplied with three different currents, resulting in nine operating conditions as shown in Table 2. The fault location in the planetary gearbox is the planet gear, with four types of faults: broken teeth, crack, surface wear, and missing teeth, as well as a normal gear, totaling five states of the planetary gear.

**Table 2:** Operating condition parameters.

Working condition number	A	B	C	D	E	F	G	H	I
Rotational speed (Hz)	20	20	20	30	30	30	40	40	40
Load (A)	0	0.4	0.8	0	0.4	0.8	0	0.4	0.8

Under a specific operating condition, sampling was conducted on a planetary gearbox with five known states. For each state, the sampling frequency was 12,800 Hz, with a duration of 1 min, resulting in 121,948 vibration monitoring points. Each sample consisted of 2,048 points, and a sliding window with a step size of 100 was used to expand the number of training and testing samples through overlapping sampling. This resulted in a training sample set of 1000 samples and a testing sample set of 200 samples. The dataset from this condition was used as the source domain, while data from another condition, collected in the same manner but with unknown fault states, served as the target domain for fault monitoring and diagnosis.

### Fault data diagram

**Fig. 5:**
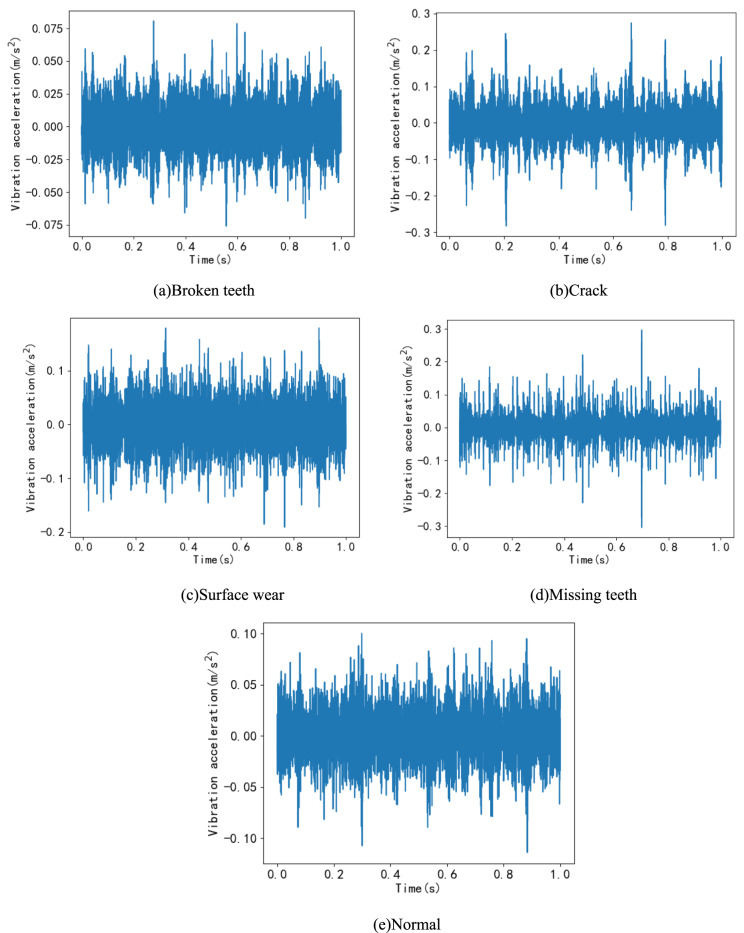
Time domain waveforms of 5 states under operating condition I.

**Fig. 6:**
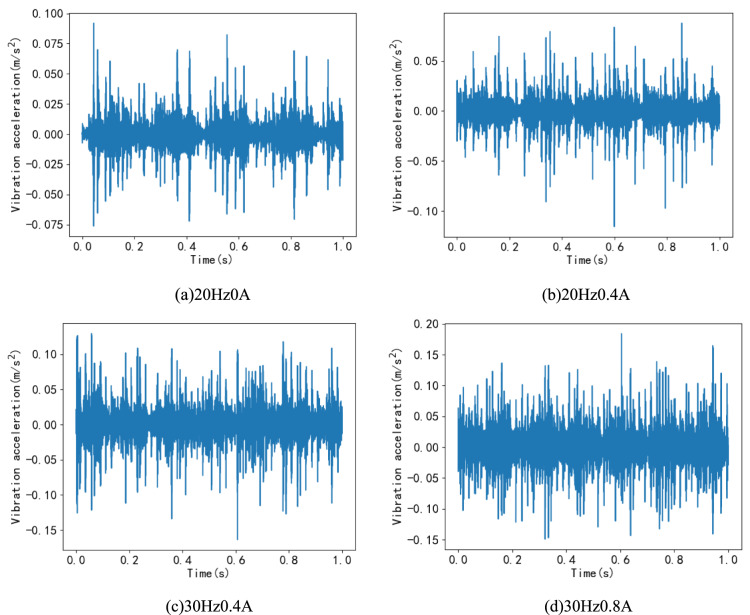
Time domain waveform diagram of missing teeth fault state.

To demonstrate the characteristics of fault data from the planetary gearbox fault diagnosis test bench, this paper, due to space limitations, only presents the time-domain signal graphs for five planetary gear states under condition number I (as shown in Fig. 5) and the time-domain waveform graphs for missing teeth fault states under conditions A, B, E, and F(as shown in Fig. 6).

Figure 5 illustrates that, under this operating condition, there are noticeable differences in the time-domain waveforms between the four fault states and the normal state, especially in the cases of broken teeth and missing teeth. Additionally, Fig. 6 demonstrates the variations in vibration acceleration even within the same missing teeth fault state under different operating conditions. The differences are particularly pronounced when the operating conditions are vastly different, such as between condition A and condition I, highlighting the complexity of fault vibrations under varying operating conditions.

### Attention mechanism comparison

**Fig. 7:**
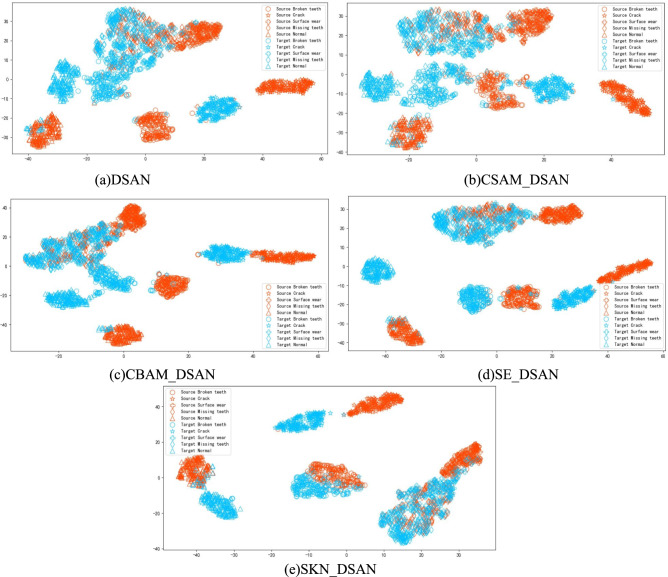
Visualization of different attention mechanism features.

To validate the responsiveness of the SKN attention mechanism with the LMMD adaptive metric criterion in the Deep Transfer Model DSAN (Deep Subdomain Adaptation Network)^27^ and to demonstrate its feature focusing capability, the SKN-DSAN model, which integrates the SKN attention mechanism, is compared with the DSAN model embedded with three different attention mechanisms: Squeeze-and-Excitation (SE), Convolutional Block Attention Module (CBAM), and Channels and Spatial Attention Module (CSM), forming the SE-DSAN, CBAM-DSAN, and CSAM-DSAN models, respectively. These models are used for comparative experiments on deep transfer tasks. Additionally, the t-distributed stochastic neighbor embedding (t-SNE) algorithm is employed to visualize the dimensionality reduction of the proposed deep features. Using operating condition G as the source domain and operating condition D as the target domain, the DSAN models embedded with different attention mechanisms are used for fault category diagnosis. The feature visualization results are shown in Fig. 7.

Figure 7 shows that the features extracted by the DSAN model with different attention mechanisms exhibit significant differences when visualized. After applying t-SNE dimensionality reduction to the deep features extracted by the DSAN model, both domains show varying degrees of overlap among certain fault type features. Specifically, there is significant overlap between broken teeth and surface wear, and some overlap between missing teeth and normal, missing teeth and surface wear, broken teeth and crack, and broken teeth and missing teeth. However, after embedding different attention mechanisms, the overlap phenomenon is reduced. The improvement with the CSAM-DSAN is not very pronounced, whereas CBAM-DSAN, SE-DSAN, and SKN-DSAN provide clearer expressions. Except for some overlap between missing teeth and surface wear, and missing teeth and normal fault features, the rest can be basically distinguished. Notably, the SKN-DSAN network exhibits only a small amount of overlap between missing teeth and surface wear fault, suggesting that the network with the embedded SKN attention mechanism is better at extracting deep fault features of the planetary gearbox, which is beneficial for fault diagnosis and classification.

To further verify that the attention mechanism, especially the SKN attention mechanism, can better extract deep features of samples in fault recognition, and to match the LMMD domain adaptation loss function in the DSAN model, the DSAN model embedded with different attention mechanisms and the features extracted by DSAN were respectively used for fault diagnosis classification with Softmax. Experiments were conducted on five random transfer tasks, and the results are shown in Table 3.

**Table 3:** The fault identification rate of the DSAN model under the attention mechanism (%).

Task	B → H	G → A	C → D	G → D	D → G
DSAN	86.3	87.9	74.0	72.9	84.0
CSAM-DSAN	88.0	90.5	93.9	77.5	88.0
CBAM-DSAN	84.0	92.0	87.7	83.5	83.3
SE-DSAN	88.8	89.3	86.1	87.4	84.4
SKN-DSAN	90.6	93.2	89.6	91.3	90.7

The fault recognition rate of the DSAN network with different attention mechanisms, as shown in Table 3, indicates that embedding the attention mechanism before the fully connected layer significantly improves the fault recognition rate obtained from deep feature classification compared to the original DSAN network. Among the various attention mechanisms, the SKN attention mechanism achieves the highest average diagnostic accuracy of 91.08%, with the smallest standard deviation of 1.33%, demonstrating better robustness in task diagnosis compared to the other three attention mechanisms. Therefore, embedding the SKN attention mechanism into the DSAN model effectively extracts key fault information and enhances the accuracy of planetary gearbox fault diagnosis.

### Deep transfer analysis

To further validate the fault diagnosis generalization and accuracy of the proposed SKN-DSAN deep transfer model under varying operating conditions, experimental analysis is conducted comparing seven classical deep learning methods: Deep Subdomain Adaptation Network (DSAN), the mainstream deep learning network CNN, Deep-Coral^28^, Deep Domain Confusion Network (DDC)^29^, Temporal Convolutional Network (TCN)^30^, improved AlexNet(AlexNe)^31^, and Transformer-CNN(Trans-CNN)^32^.

**Fig. 8:**
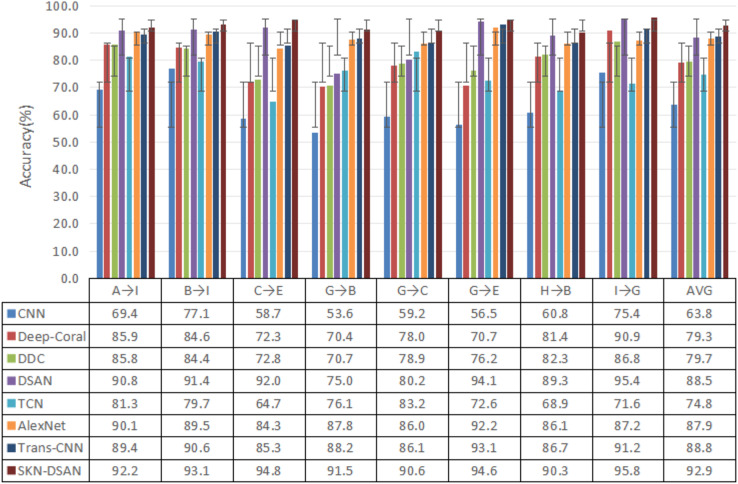
Results of experiments on different models under variable operating conditions.

In the aforementioned methods, the same convolutional neural network structure is used, with parameter settings consistent with those of the proposed method. The sample dataset is constructed using the same sampling approach as the proposed method. Testing is conducted on the same eight variable condition transfer tasks, with each method being tested 10 times and the average value taken. The comparison results are shown in Fig. 8.

As shown in Fig. 8, the fault identification accuracy of the SKN-DSAN model is higher than that of seven classic deep learning models. Although CNN has feature extraction capabilities, under varying working conditions, the fault feature distribution exhibits significant differences. Models trained with features extracted from the source domain cannot adapt to fault recognition in the target domain under varying working conditions. In contrast, the TCN network, which extracts temporal features from vibration signals, the improved AlexNet, and the Trans-CNN network which bases on transformer and convolutional neural network, as well as the other three deep transfer learning models that adaptively match the distribution differences between the source and target domains, all achieve higher fault identification accuracy than CNN. The SKN-DSAN and DSAN models use the LMMD domain adaptation metric to match the conditional distribution differences between the two domains. Compared to the MMD metric used by DDC and the Coral metric used by Deep-Coral, which focus on the global domain’s marginal distribution, LMMD has stronger adaptive capabilities, resulting in higher fault identification rates. The proposed SKN-DSAN adds an SKN attention mechanism before the fully connected layer in DSAN, which adaptively adjusts the weight of the neuron response values of the subdomain feature information in both domains. This mechanism not only uses the domain-specific neuron receptive field features of training samples as a reference but also effectively captures the deep internal connections between the source and target domain subdomains. By assigning higher weights to critical features around the neuron’s adaptive response values, it selects key fault features for transfer adaptation, outperforming traditional methods that rely solely on maximum or average pooling combined with fully connected layers to merge information, which leads to the failure to recognize key information. The model achieves a minimum accuracy of 90.3% across eight transfer tasks, with an average accuracy improvement of 4.4%, 5.0%, and 4.1% compared to DSAN, AlexNet, and Trans-CNN, respectively, which are models with relatively high fault recognition rates. Additionally, it exhibits the shortest standard deviation of accuracy, at 1.92%. These results indicate that the SKN-DSAN model demonstrates superior fault diagnosis accuracy and generalization capability under varying working conditions compared to CNN, TCN, the improved AlexNet deep learning model, and four deep transfer learning models: DDC, Deep-Coral, DSAN, and Trans-CNN.

### Visualization of deep transfer results

**Fig. 9:**
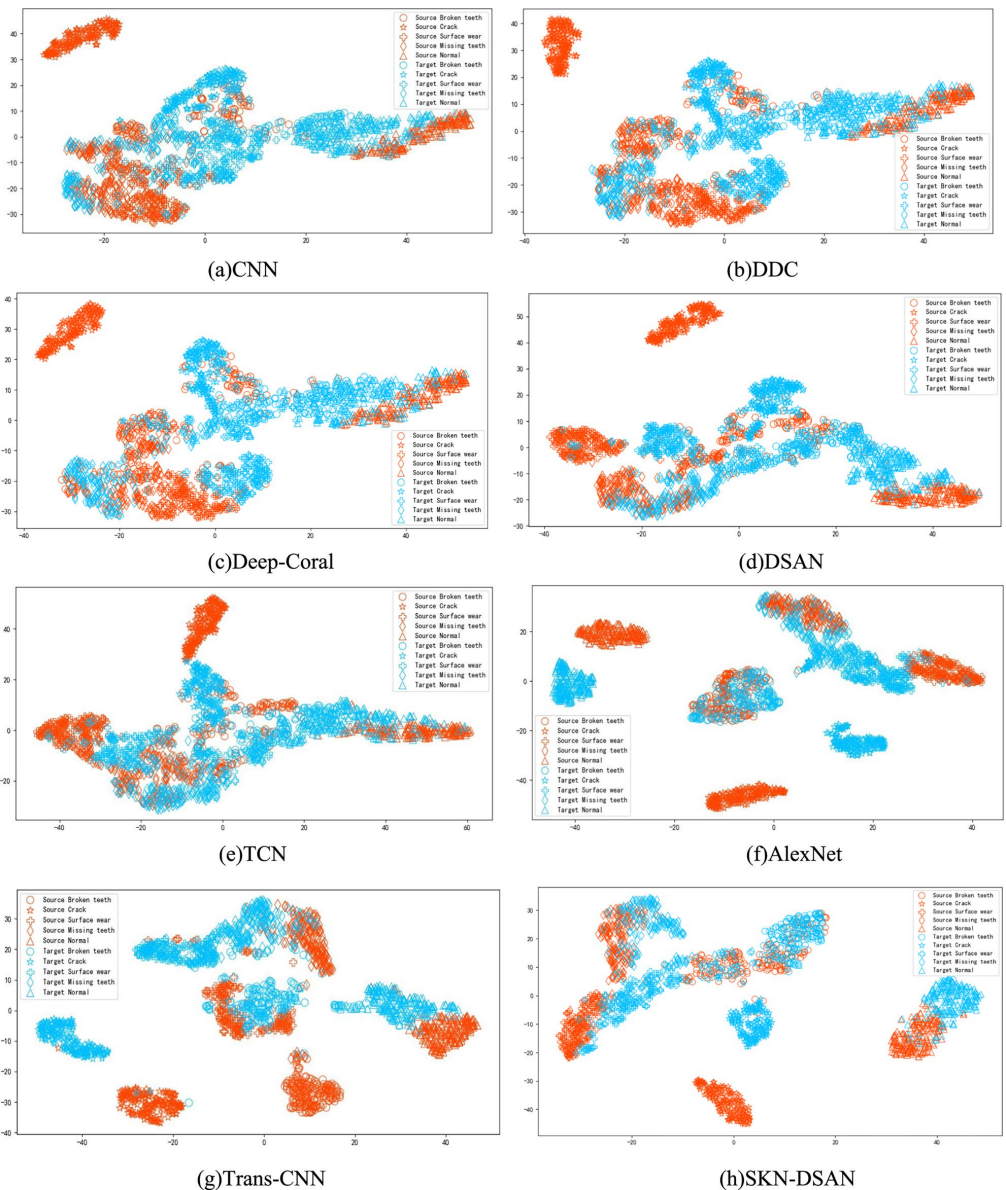
Visualization results of features using different methods.

To more intuitively illustrate the feature alignment capability of the SKN-DSAN model in deep transfer learning, the feature distributions under different operating conditions are visualized. Taking the transfer task G → B as an example, t-SNE visualization is performed, as shown in Fig. 9.

According to Fig. 9, (a) shows significant overlap among broken teeth, crack, surface wear, missing teeth, and normal conditions, with particularly severe overlap between broken teeth and crack, as well as surface wear. In (b), (c), (d), and (e), there is varying degrees of overlap among the four types of faults: broken teeth, missing teeth, crack, and surface wear. In (f), there is minor overlap between missing teeth and crack, missing teeth and surface wear, missing teeth and broken teeth, as well as between broken teeth and surface wear, and missing teeth. The (g) exhibits minor overlap between missing teeth and surface wear, missing teeth and broken teeth, broken teeth and normal conditions, as well as between broken teeth and surface wear, and missing teeth. However, in (h), only minor overlap is observed between broken teeth and crack, missing teeth and surface wear, and broken teeth, with clear clustering boundaries, indicating almost complete separation of fault categories. This suggests that the SKN attention mechanism adapted to the LMMD metric not only focuses on the relationships within subdomains of the same category across different domains but also emphasizes the extraction of key features. The combination of these two aspects in feature learning assigns higher weights to features with smaller distances, thereby better aligning subdomain features between the source and target domains, demonstrating strong clustering capability.

**Fig. 10:**
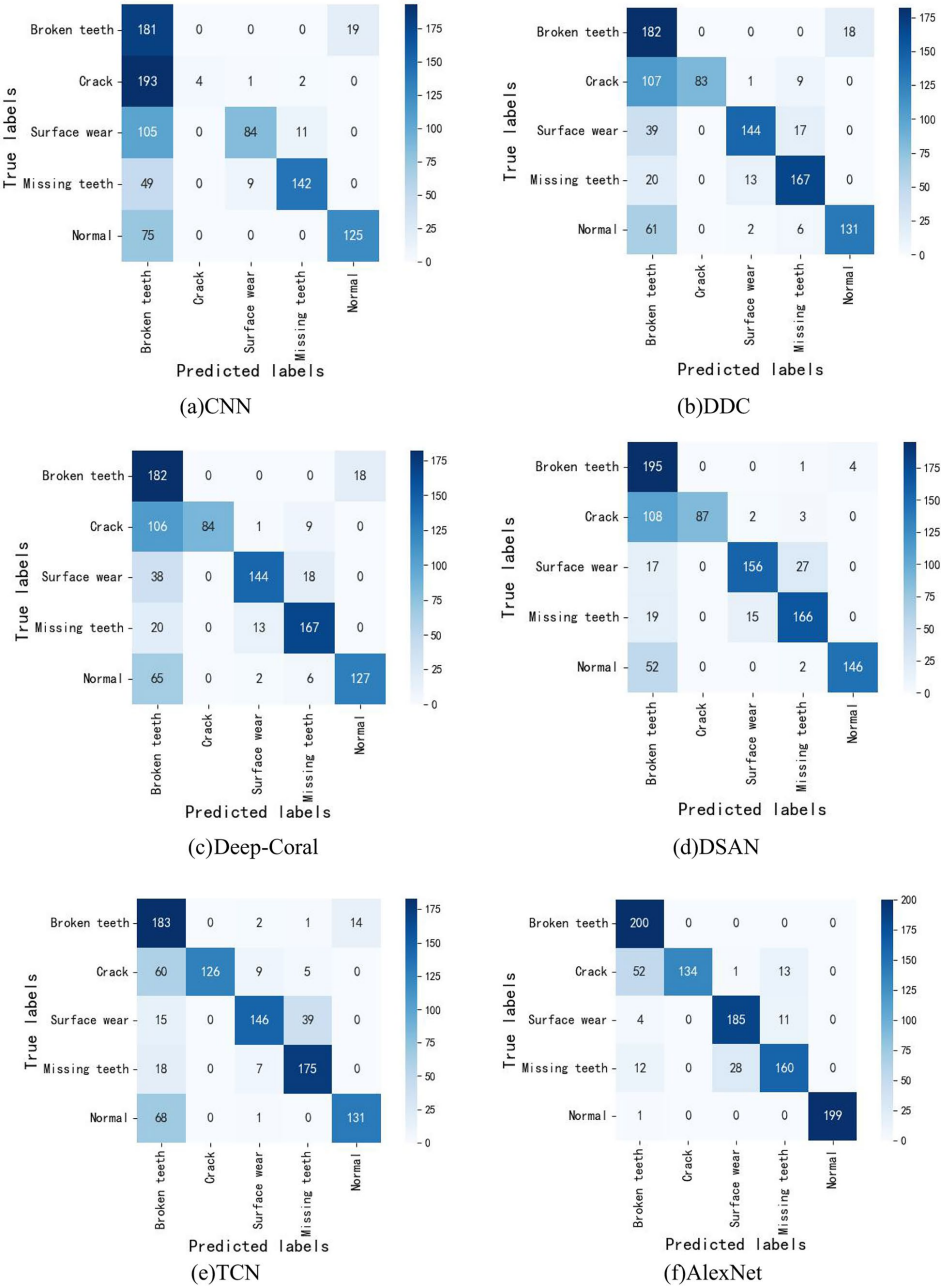

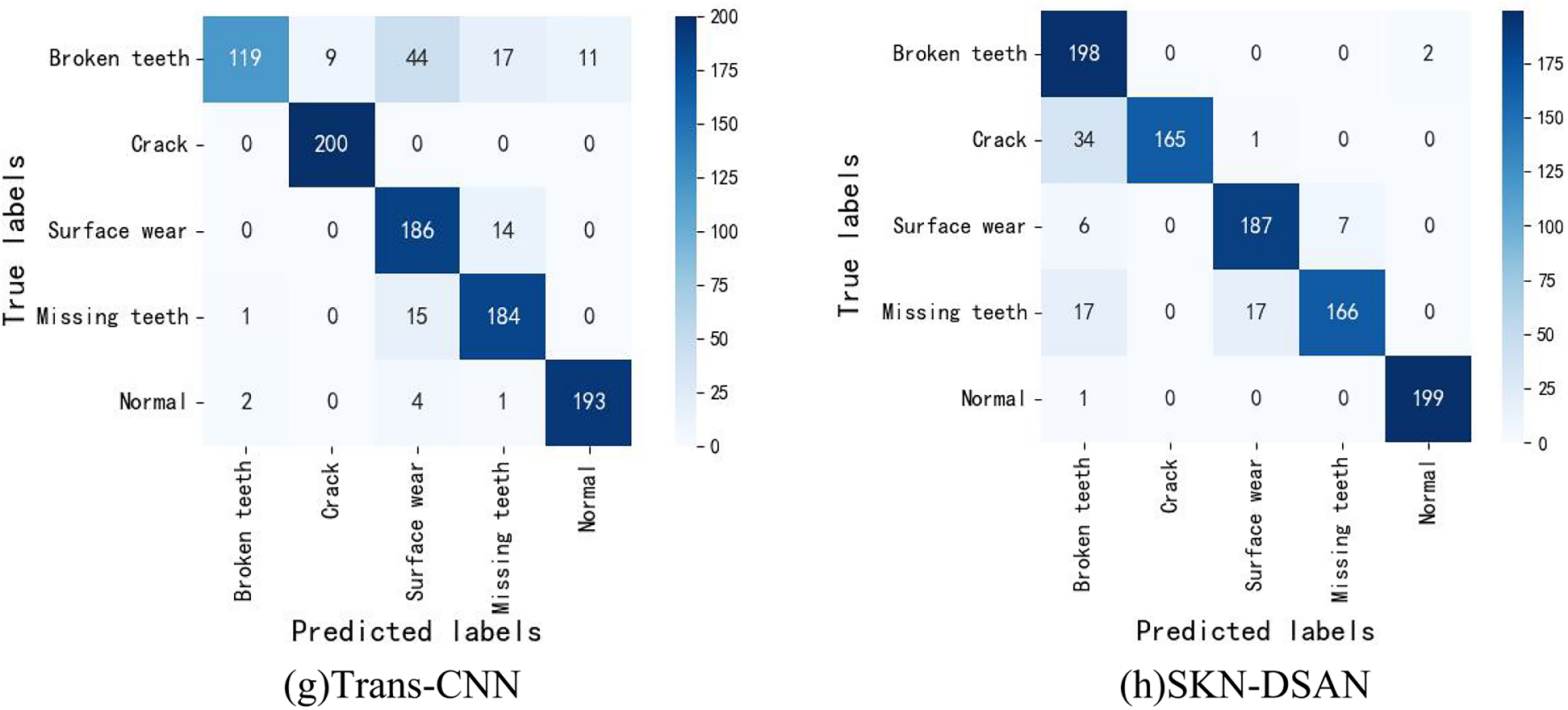
The confusion matrix results of different models.

To more clearly analyze the diagnostic effectiveness of the eight methods, a confusion matrix is introduced to display the fault diagnosis results for 1000 sample data, as shown in Fig. 10. From Fig. 10, it can be seen that in (a), (b), (c), (d), (e), and (f), a large number of crack fault type samples are misclassified, particularly in (a), where crack fault types are almost entirely misclassified as broken teeth. In (b), (c), and (d), more than 50% of crack fault types are incorrectly classified as broken teeth. There are also numerous classification errors in the surface wear, missing teeth, and normal categories, with only the broken teeth fault type having acceptable classification accuracy. In (g), over 80% of broken teeth faults are misclassified. In contrast, in (f), aside from a small portion of misclassifications in the crack and missing teeth categories, the normal, broken teeth, and surface wear categories are almost accurately classified. Notably, out of 200 broken teeth samples, only 2 are incorrectly classified as normal, and out of 200 normal samples, only 1 is misclassified as broken teeth, achieving an almost 100% fault diagnosis accuracy. This demonstrates that the proposed SKN-DSAN model exhibits a high recognition capability for various fault types under varying working conditions.

## Conclusions

To address the issues of complex fault characteristics in planetary gearboxes, difficulty in capturing fault features under varying working conditions, imbalanced fault data distribution, and poor diagnostic performance, a fault diagnosis method based on SKN attention mechanism and deep transfer learning is proposed. Experimental validation has led to the following conclusions:

1. The SKN_DSAN, DSAN, and three new deep transfer models formed by embedding three different attention mechanisms into the deep transfer model DSAN were compared using t-SNE visualization under varying operating conditions for deep transfer fault diagnosis. It was found that the SKN attention mechanism has a more intuitive ability to aggregate fault categories. Meanwhile, in fault recognition and diagnosis under five varying operating conditions, the SKN_DSAN model achieved an average diagnostic accuracy of 91.08%, with a standard deviation of only 1.33%. The SKN attention mechanism demonstrates a stronger ability to extract key fault information.
2. SKN_DSAN and seven mainstream deep learning models were used to perform fault diagnosis on a planetary gearbox under eight different working condition tasks. The comparison revealed that the model achieved a minimum fault recognition rate of 90.3%, with a standard deviation of 1.92%. The SKN attention mechanism, combined with the LMMD domain adaptation metric, more effectively selects key fault features for transfer adaptation. Additionally, t-SNE visualization of the transfer task shows that the model better aligns similar features, and the confusion matrix indicates that the model has a more generalized recognition ability for various faults under varying working conditions.

## Data Availability

The datasets generated and/or analysed during the current study are not publicly available due the data also forms part of an ongoing study, but are available from the corresponding author on reasonable request.
